# Trends in Chronic Myeloid Leukemia Mortality by Race, Age, and Gender in the United States Between 1999 and 2020

**DOI:** 10.7759/cureus.110083

**Published:** 2026-06-01

**Authors:** Saad Rashid, Abhinav Kakuturu, Zeeshan Muzammil, Heena Parkash

**Affiliations:** 1 Department of Internal Medicine, Javon Bea Hospital, Rockford, USA; 2 School of Medicine, Rosalind Franklin University of Medicine and Science, North Chicago, USA

**Keywords:** chronic myeloid leukemia (cml), demographic trends, healthcare disparities, hematologic malignancy, tyrosine kinase inhibitors (tki)

## Abstract

Background and objective

Chronic myeloid leukemia (CML) represents a significant portion of leukemia cases annually in the United States. Despite advances in treatment, disparities in CML mortality persist across different demographic groups, particularly by race, age, sex, and social determinants of health. This study explores mortality trends by race, age, and gender in CML from 1999 to 2020 in the United States.

Methods

Mortality data for CML (ICD-10 code C92.1) were analyzed from the CDC Wonder Database (1999-2020). Age-adjusted mortality rates (AAMRs) were calculated per 100,000 persons and stratified by sex, race (White and African American), and adult age groups.

Results

While overall CML mortality rates declined after the introduction of tyrosine kinase inhibitors (TKIs) in 2001, disparities persist. Older adults, males, and African Americans exhibit higher mortality rates compared to their counterparts. African Americans’ AAMR decreased from 0.7 in 1999 to 0.35 by 2008 (annual percent change (APC) of -8.5%, p < 0.01). White Americans had a larger reduction in AAMR, from 0.65 in 1999 to 0.3 by 2007 (APC of -10.0%, p < 0.01). Age-specific analyses highlighted a decrease in mortality across adult age groups. The oldest group, aged ≥85 years, had an AAMR of 5.25 in 1999, which decreased to 4.7 by 2020 but remained significantly higher than other age groups.

Conclusions

Despite the effectiveness of TKIs, significant disparities in CML mortality remain, particularly among older adults, males, and African Americans. Addressing these disparities requires a multifaceted approach, including better access to care, increased representation in clinical trials, and tailored interventions for each demographic group, which is critical to reducing these disparities and improving outcomes for all patients.

## Introduction

Chronic myeloid leukemia (CML) is a myeloproliferative neoplasm that accounts for approximately 15% of all adult leukemias in the United States [1]. The global incidence of CML in 2023 is estimated to be one case per 100,000 people, with the United States presenting nearly 9,000 new CML diagnoses annually [2]. The introduction of tyrosine kinase inhibitors (TKIs) has transformed CML from a once-fatal disease into a chronic, manageable condition for the majority of patients. However, the extent to which these advancements in treatment have translated into reduced mortality across different demographic groups remains unclear and understudied.

While overall cancer-related mortality in the United States has significantly declined since the 1990s, driven by advancements in earlier detection, improved treatment, and reductions in tobacco use, persistent inequities remain [3]. These disparities are especially evident across racial, sex, geographic, and socioeconomic groups. These ongoing inequities highlight the crucial role of social determinants of health (SDOH), such as access to healthcare, education, income, and environmental factors, in shaping health outcomes [4].

Disparities in cancer mortality have been well documented in several solid tumors and hematologic malignancies, such as chronic lymphocytic leukemia [5]. However, limited research has focused specifically on CML. With nearly 9,000 new CML diagnoses annually in the United States, understanding how age, race, and gender intersect with mortality risk is important for guiding equitable care strategies in CML [2].

This study examines trends in CML-related mortality in the United States from 1999 to 2020 using CDC WONDER mortality data, with a focus on disparities across age, race, and sex. It aims to assess whether improvements in treatment outcomes have been equitably distributed by (1) characterizing overall mortality trends and (2) evaluating demographic differences through predefined subgroup analyses to identify persistent disparities across population groups.

## Materials and methods

Study design

This is a retrospective cohort study analyzing trends in CML-related mortality in the United States from 1999 to 2020. The study evaluates mortality differences across age, sex, and race.

Data sources and collection

The CDC WONDER database was used to access national mortality data for CML, identified by ICD-10 code C92.1, from 1999 to 2020. The dataset included publicly available records of deaths in which CML was listed as the underlying cause. Analyses were conducted using CDC WONDER’s built-in stratification variables, including age, sex, and race/ethnicity, without additional case-level refinement beyond database-defined filters. The CDC WONDER database is widely validated, with mortality data undergoing standardized collection and quality assurance processes, including verification against medical death certificates.

Study population

The study population included individuals with CML-related deaths from 1999 to 2020. Analyses were restricted to individuals aged ≥45 years due to the low incidence of CML-related mortality in younger populations, which limits statistical stability. Mortality data were stratified by sex (male and female), race (White and African American), and age groups (45-54, 55-64, 65-74, 75-84, and ≥85 years).

Statistical analysis

Age-adjusted mortality rates (AAMRs) per 100,000 persons were calculated for CML-related deaths in the United States from 1999 to 2020. AAMRs were derived by dividing the number of CML-related deaths in each group by the corresponding population and directly standardizing to the 2000 US Standard Population.

Temporal trends in CML mortality were evaluated using Joinpoint Regression Software (version 4.9.0.0, National Cancer Institute, Bethesda, MD, USA), which is freely available for academic use. The model identified changes in trend slopes and estimated annual percent change (APC) for each segment across the study period. The average APC (AAPC) was calculated to summarize overall long-term trends by averaging APCs across the full time interval. The number of joinpoints was constrained between 1 and 4 to allow for up to four changes in trend over time. The grid search method (2, 2, 0) was used to select the optimal model, and permutation tests assessed statistical significance, with p < 0.05 considered significant.

Pairwise comparisons between demographic groups (age, race, and sex) were conducted using two-tailed t-tests to identify differences in mortality rates. No adjustments were made for multiple comparisons, as analyses were exploratory and intended to describe group-level differences. All analyses were conducted using publicly available data from CDC WONDER. Data extraction used ICD-10 code C92.1 as the underlying cause of death, with stratification by age, sex, and race/ethnicity as defined within the database. AAMRs were calculated using the direct method based on the 2000 US Standard Population. Given the ecological nature of CDC WONDER data, multivariable regression adjusting for individual-level factors (e.g., comorbidities, treatment exposure, and socioeconomic status) was not possible. Accordingly, analyses were limited to stratified descriptive statistics and Joinpoint regression-based trend assessment.

Data visualization

Statistical analyses were performed using Joinpoint Regression Software and RStudio (version 4.0.3; RStudio, PBC, Boston, MA, USA) for data visualization. Mortality trend figures were generated to illustrate changes in CML mortality rates across subgroups. Temporal trends were assessed for statistical significance using Wald’s chi-square test in Joinpoint and R environments.

## Results

Total population

Between 1999 and 2020, 26,329 deaths occurred due to CML or with CML as the underlying cause of death in the United States. Overall, the AAMR decreased by 50% during this period. Table 1 provides a stratified breakdown of deaths by sex, race, and age, with AAMR analysis performed using Joinpoint regression.

**Table 1:** Demographic characteristics of deaths due to CML in the US, 1999-2020 AAMR, age-adjusted mortality rate; AAPC, average annual percent change; CML, chronic myeloid leukemia

Category	Subgroup	# of CML deaths (%)	AAMR per 100,000	AAPC trend (95% CI)
Overall	All	26,329 (100%)	-	-
Gender	Male	14,772 (56.1%)	0.80 (1999) to 0.40 (2007)	-3.60 (-3.99, -3.19)
	Female	11,557 (43.9%)	0.50 (1999) to 0.20 (2007)	-4.91 (-8.29, -1.40)
Race/ethnicity	African American	2,250 (8.5%)	0.78 (1999) to 0.36 (2007)	-4.48 (-5.78, -3.02)
	White American	21,019 (79.8%)	0.65 (1999) to 0.30 (2007)	-3.84 (-4.34, -3.33)
Age group	45-54 years	2,278 (8.7%)	0.60 (1999) to 0.25 (2004)	-6.91 (-8.72, -4.40)
	55-64 years	3,361 (12.8%)	1.00 (1999) to 0.40 (2020)	-5.30 (-6.52, -4.09)
	65-74 years	5,082 (19.3%)	2.25 (1999) to 0.80 (2008)	-5.44 (-6.44, -4.25)
	75-84 years	7,513 (28.5%)	2.40 (1999) to 3.80 (2006) to 2.25 (2011)	-0.31 (-0.91, 0.31)
	≥85 years	5,620 (21.3%)	5.25 (1999) to 4.70 (2020)	-0.30 (-0.90, 0.38)

Ten-year age groups

When comparing overall CML-associated mortality from 1999 to 2020, the AAMR was highest in the 85+ age group, followed by the 75-84, 65-74, 55-64, and 45-54 age groups. Furthermore, those in the 85+ year age group demonstrated a decrease in AAMR from 5.25 in 1999 to 4.70 in 2020. In the 75-84 age group, AAMR initially increased from 2.40 in 1999 to 3.80 in 2006. Subsequently, the AAMR decreased from 3.80 in 2006 to 2.25 in 2011. Notably, APC increased by +19.5% from 2003 to 2006 (p < 0.05) and decreased by 10.4% from 2006 to 2011 (p < 0.05). In the 65-74 age group, AAMR decreased from 2.25 in 1999 to 0.80 in 2008. Additionally, the APC declined by 11.0% between 1999 and 2007 (p < 0.05). For the 55-64 age group, AAMR decreased from 1.00 in 1999 to 0.40 by 2020, with an APC decrease of -11.9% (p < 0.05). For individuals aged 45-54, the AAMR declined from 0.60 in 1999 to 0.25 in 2004. Additionally, APC decreased by -21.2% from 1999 to 2004 (p < 0.05).

**Figure 1:**
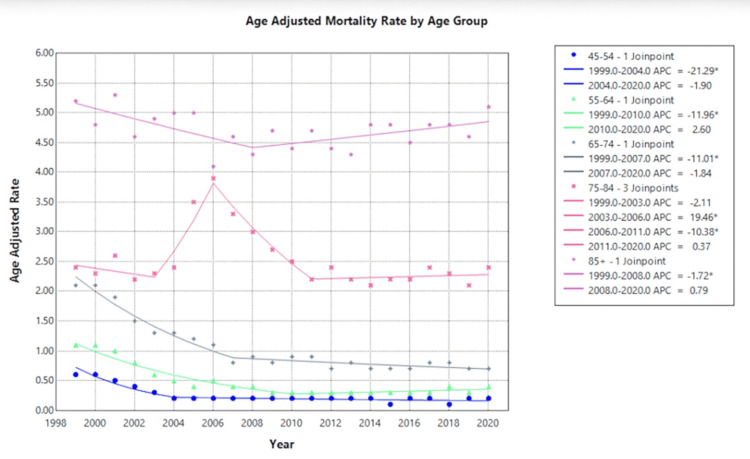
AAMR by age group AAMR per 100,000 secondary to CML between 1999 and 2020 with respect to five 10-year age groups: ages 45-54, 55-64, 65-74, 75-84, and 85+. Asterisks (*) indicate statistically significant joinpoints, where a significant change in the APC occurred. AAMR, age-adjusted mortality rate; APC, annual percent change

From 1999 to 2020, the overall AAPC for those aged 85+ was -0.30% (95% CI = -0.90 to 0.38). For the 75-84 age group, the overall AAPC from 1999 to 2020 was -0.31% (95% CI = -0.91 to 0.31). In the 65-74 group, AAPC decreased by 5.44% (95% CI = -6.44 to -4.25). For those aged 55-64, the AAPC during this time was -5.30% (95% CI = -6.52 to -4.09). Finally, for those aged 45-54, the AAPC decreased by 6.91% (95% CI = -8.72 to -4.40) from 1999 to 2020. Figure 1 visualizes these findings.

Sex

Reduction in CML-associated mortality differed with respect to sex. In women, the AAMR decreased from 0.50 in 1999 to 0.20 by 2007. The APC declined by 10.6% from 1999 to 2008 (p < 0.01). The overall AAPC from 1999 to 2020 was -4.91% (95% CI = -8.29 to -1.40).

**Figure 2:**
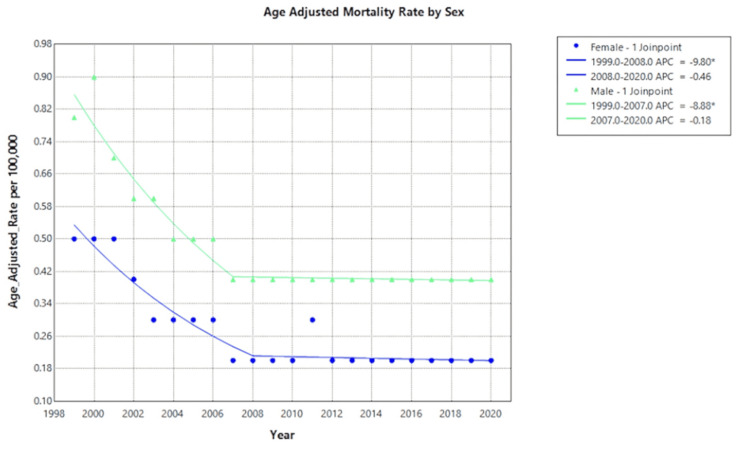
AAMR by sex AAMR per 100,000 secondary to CML between 1999 and 2020 with respect to sex (male and female). Separated according to sex (male and female). Asterisks (*) indicate statistically significant joinpoints, where a significant change in the APC occurred. AAMR, age-adjusted mortality rate; APC, annual percent change

In males, the AAMR decreased from 0.80 in 1999 to 0.40 by 2007. The overall AAPC from 1999 to 2020 was -3.60 (95% CI = -3.99 to -3.19). The APC declined by 8.9% from 1999 to 2007 (p < 0.01). Figure 2 visualizes these findings.

Race

**Figure 3:**
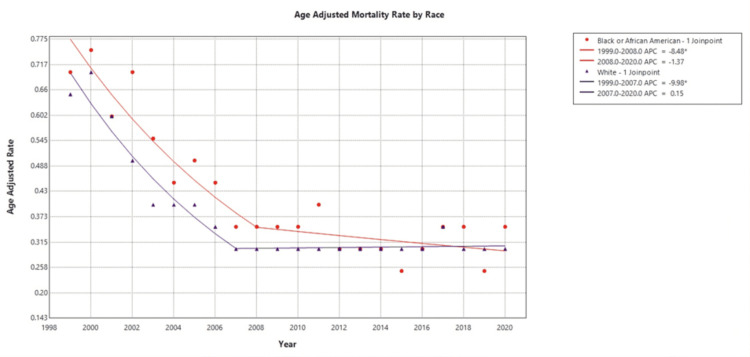
AAMR by race AAMR per 100,000 secondary to CML between 1999 and 2020 with respect to race (African American and White American). Asterisks (*) indicate statistically significant joinpoints, where a significant change in the APC occurred. AAMR, age-adjusted mortality rate; APC, annual percent change

When adjusting for race, the rate of AAMR decreased by varying degrees. In African Americans, the AAMR decreased from 0.78 in 1999 to 0.36 by 2007 (APC of -8.5%, p < 0.01). In comparison, White Americans were found to have a reduction in AAMR from 0.65 in 1999 to 0.3 by 2007 (APC -10.0%, p < 0.01). From 1999 to 2020, the AAPC decreased overall for African Americans by -4.48% (95% CI = -5.78 to -3.02). For White Americans, the AAPC decreased by -3.84% (95% CI = -4.34 to -3.33). Figure 3 visualizes these findings.

## Discussion

The introduction of TKIs has significantly improved survival outcomes in CML. TKIs specifically target the BCR::ABL1 fusion protein and inhibit its proliferative effects [6]. In our study, according to the CDC WONDER database, 1,788 patients died of CML in 1999 compared to 1,233 in 2003. This coincides with the approval of TKIs for CML treatment in 2001. Despite these advancements, notable differences in survival outcomes persist across demographic groups. Interestingly, the overall mortality rates remained relatively stable between 2003 (1,233 deaths) and 2020 (1,256 deaths). This suggests that while treatment advances have improved survival, the persistence of disparities driven by underlying SDOH may have slowed improvements in CML-related mortality. Mortality still differs according to age, race, and sex, even with the availability of TKIs.

These findings should be interpreted as descriptive, population-level associations rather than causal relationships, given the ecological nature of the data and absence of individual-level clinical variables. Temporal trends in mortality should also be interpreted in the context of potential changes in diagnostic practices, coding accuracy, and competing causes of death over the study period, which may have influenced observed mortality patterns despite the use of standardized age-adjusted methods and Joinpoint regression.

Disparities by race

Our study found that African Americans experienced higher mortality rates related to CML compared to White Americans. Specifically, African American patients had an AAMR of 0.78 in 1999, which decreased to 0.36 in 2007, as compared to an AAMR of 0.65 in 1999, which decreased to 0.30 by 2007 for White Americans (p < 0.05). The AAPC over this period was -4.48 for African Americans and -3.84 for White Americans (p < 0.05). While both groups displayed a decrease in mortality over time, African Americans displayed higher mortality rates than White Americans. However, more recent data from 2017 to 2020 indicate a notable shift in this trend, as CML mortality rates among White Americans are now surpassing those faced by African Americans. While it is too early to draw definitive conclusions, this divergence may signal early progress in addressing long-standing disparities in cancer care. However, this post-2017 convergence should be interpreted cautiously, given the ecological nature of the data and potential instability of estimates in smaller subgroup analyses during later time periods.

The overall trend mirrors long-standing broader cancer mortality patterns, where African Americans experience higher death rates from various cancers, including colon, breast, uterine, cervical, lung, and prostate cancers [7]. These poorer cancer outcomes are due to a complex mix of social, economic, biological, and systemic factors. However, observed racial differences in mortality are likely multifactorial and cannot be attributed to biological differences alone. Alternative explanations include differences in access to cancer care, timing of diagnosis, socioeconomic barriers, insurance status, geographic distribution of specialty care, and differences in treatment adherence and persistence with TKI therapy. The small population subset also limits generalizability, as small changes in this population may greatly affect results.

Biologic and genomic factors contribute to racial disparities in mortality. A study by Brown et al. demonstrated that African American patients are more likely to exhibit whole-genome duplications, a genomic event that enhances metastasis and aggressive disease, than White Americans [8]. This has been observed across breast, endometrial, and lung cancers and is associated with less favorable prognoses. For example, many single-nucleotide polymorphisms (SNPs) and copy number variations are associated with racial diversity [9]. Further studies are ongoing to evaluate SNPs among different races, which may form the basis for future targeted therapies. In a study conducted by Myer et al., they found that patients of African descent had significantly higher KRAS mutations than patients of other descent [10]. These mutations are resistant to certain therapies and correlate with a more aggressive disease course [11]. While no similar analyses in CML have been completed, it is plausible that genetic variations, such as differences in cytokine profiles, inflammatory markers, or drug metabolism, could influence disease progression or treatment response in African American patients. Overall, genetic mutations contribute significantly to survival disparities in malignancy among races and require further evaluation in CML.

Implicit racial bias contributes to disparities in cancer treatments. A study conducted by Penner et al. evaluated 18 non-African American oncologists and 118 African American patients. As predicted, oncologists with higher implicit racial bias had shorter interactions, increased difficulty with patients remembering the contents of the interaction, and decreased patient confidence in recommended treatments [12]. These communication gaps may ultimately affect adherence and treatment efficacy.

Structural inequities further compound survival differences. African American patients often encounter delayed diagnosis and treatment initiation. Moreover, this population remains significantly underrepresented in TKI-related clinical trials, limiting the applicability of safety and efficacy findings to their care [13]. The study by Reddy et al. demonstrated that Black and Hispanic races are consistently underrepresented among studies evaluating TKIs [14]. Community-level resource deficits also influence mortality trends for African Americans. For example, African American communities are disproportionately located in areas with lower concentrations of oncologists and limited access to advanced cancer care, leading to delays in care initiation [15].

To address these disparities, several actionable strategies can be employed. Expanding access to hematology/oncology services in underserved communities is essential. Strategies for expanding access include the introduction of telemedicine programs to allow healthcare providers to provide care to underserved regions. Incentivizing hematology-oncology providers through programs such as loan forgiveness to practice in health professional shortage areas may further enhance this. Reducing financial and logistical barriers to TKI therapy can improve access and adherence to medications. Examples include improved co-pay assistance, mail-order pharmacies, and donation programs. For example, the Leukemia & Lymphoma Society has begun partnering with RemediChain to accept donations of unopened, unexpired medications and match the donations for vulnerable patients in need. Additionally, mandatory implicit bias training for hematology-oncology providers may reduce bias encountered by African American patients. Lastly, improving diversity in clinical trials is vital to more equitable care. Enhancing survival outcomes in African American patients with CML will require a multi-pronged approach that includes health system reform, targeted outreach, further research, and culturally competent care.

While biologic and genomic differences have been proposed in cancer disparities research, their direct contribution to CML-specific mortality differences remains unproven and should be interpreted as a hypothesis rather than an established mechanism.

Disparities by age

Just as race plays a significant role in influencing CML-related survival, age remains one of the most impactful factors in determining mortality rates. Our study analysis reveals a clear age-related increase in CML mortality, with individuals aged ≥85 years experiencing the highest mortality rates. Specifically, the AAMR for individuals aged 85+ was 5.25 in 1999, compared to 2.40, 2.25, 1.00, and 0.60 for patients aged 75-84, 65-74, 55-64, and 45-54, respectively (p < 0.05). The oldest age group also showed a less pronounced decline in mortality relative to other age groups, with an AAPC from 1999-2020 of -0.30, as compared to -0.31, -5.44, -5.30, and -6.91 in patients aged 75-84, 65-74, 55-64, and 45-54, respectively (p < 0.05). These trends demonstrate that as age increases, mortality rates from CML also rise, while the decline in mortality over time becomes less pronounced.

Similar age-related trends have been reported in other malignancies, such as lung cancer and acute myeloid leukemia (AML). A study by Pham et al. found that patients with stage III/IV lung cancer aged 80 years and older had significantly shorter median survival than patients aged 60 years and younger, with a 28% increased adjusted mortality risk (p = 0.005). This was associated with a reduced probability of receiving cancer treatment despite controlling for sex, performance status, comorbidities, and histological type [16]. In AML, patients aged 75 years and older have a life expectancy of less than one year, with only 20% of patients surviving beyond one year after diagnosis and fewer than 4% surviving for three years [17].

The higher mortality observed in older CML patients is likely multifactorial. Aging is associated with physiological changes, such as decreased functional reserves, increased comorbidities, and reduced life expectancy [18]. Additionally, functional and social factors related to aging contribute to poor outcomes [17]. Frailty, which becomes more pronounced with age, leads to decreased physical activity, poor nutrition, increased stress, and decreased organ function [19]. However, these changes occur at different rates among individuals. Interestingly, in non-small cell lung cancer, despite concerns that older patients would tolerate chemotherapy poorly and thus receive less treatment than younger patients, Früh et al. showed that overall toxicity rates were similar between different age groups [20].

Currently, no gold standard exists for assessing treatment fitness in older adults with CML, leading to subjectivity in therapeutic decisions. Geriatric assessments that evaluate physical and cognitive functions may assist in predicting chemotherapy toxicity and survival [21]. In CML, older patients are more prone to increased toxicity from TKIs, such as imatinib. For example, fluid retention is more common after the age of 65 [22]. Despite these complexities, improving access to and adherence to appropriate therapies could significantly improve survival outcomes in older patients with CML. A population-based study conducted by Daskalakis et al. in Switzerland examined the survival outcomes of different age groups with CML before and after TKI initiation. In the most recent study period (2013-2017), the five-year relative survival rate for elderly patients was only 53%, compared to 89% in younger patients [23]. This study emphasized that TKI initiation had a more substantial impact on survival in older patients than in younger adults. Ultimately, while age-related factors such as frailty, comorbidities, and treatment toxicity contribute to higher mortality rates in older patients, the complex interplay between these factors underscores the need for tailored treatment approaches.

A variety of strategies may be employed to reduce mortality in older adults with CML. One approach involves adjusting treatment regimens and goals based on patient-specific factors. For example, second-generation TKIs such as nilotinib and dasatinib have demonstrated greater potency than imatinib in achieving cytogenetic and molecular responses [24]. This may allow for achieving a deeper molecular response, earlier remission, and the potential for earlier treatment discontinuation. In selected older adults who can tolerate these agents, initiating treatment with more potent TKIs may offer survival advantages. However, for frailer patients, shared decision-making is critical to aligning treatment intensity with individual health status and preferences. In such cases, focusing on disease control rather than deep molecular remission may reduce treatment-related toxicity and improve quality of life.

Improving the inclusion of older adults in clinical trials is essential. Historically, this population has been underrepresented in oncology studies, including those evaluating TKIs. This has limited the generalizability of trial data to real-world elderly patients [25]. This exclusion contributes to uncertainty regarding optimal dosing, toxicity management, and treatment duration in older adults. Increasing the enrollment of elderly patients, especially those with comorbidities, would generate age-specific evidence to inform and create personalized treatment protocols.

Disparities by gender

While age plays a significant role in CML mortality, gender also influences survival trends. In our study, males experienced consistently higher mortality rates due to CML compared to females. Specifically, the AAMR for males declined from 0.80 in 1999 to 0.40 in 2007, whereas for females it declined from 0.50 to 0.20 during this period (p < 0.05). Although both groups experienced improvement, females had a steeper decline in mortality with an AAPC from 1999 to 2020 of -4.91, as compared to -3.60 in males (p < 0.05). This data demonstrates a persistent gender gap in CML outcomes despite overall advances in treatment.

The reasons for gender disparities in CML mortality are multifactorial, involving both biological and behavioral differences [26]. Behaviorally, differences in smoking, alcohol use, diet, cancer screening, and healthcare resource use are considered primary contributors to this disparity [26]. It is also important to note that differences in healthcare utilization patterns, comorbidity profiles, and treatment adherence may partially explain observed disparities, rather than sex-specific biological effects alone. Men are more likely to engage in higher levels of alcohol and tobacco use, both of which contribute to a higher incidence of cancer [27]. Men are also less likely to undergo regular cancer screening or seek healthcare [28]. These patterns may contribute to delayed diagnosis at advanced stages and reduced adherence to TKI therapy, contributing to higher mortality.

Biological differences also contribute to outcome differences. Jackson et al. evaluated differences in cancer occurrence and found that male predominance for most cancers remained even after adjusting for these risk behaviors and carcinogenic exposures [26]. This suggests that sex-related biological mechanisms are major determinants of differences in the risk of cancer at most shared anatomic sites. Several biological differences likely contribute to the higher cancer susceptibility observed in men. Hormonal factors, such as higher testosterone levels in men, may promote cell growth and contribute to cancer incidence [29]. Immunologically, women exhibit stronger innate and adaptive immune responses, leading to faster pathogen clearance, reduced susceptibility to cancer, and potentially influencing disease control [26]. For example, women mount more robust immune responses to oncogenic infections, such as hepatitis B and human papillomavirus, which may contribute to lower risks of liver and oropharyngeal cancers, respectively [30]. Metabolomics also plays an important role in this process. Studies have shown that males have higher levels of IL-6, an inflammatory marker that may contribute to the progression of certain cancers. Epigenetic mechanisms also play key roles. For example, age-related mosaic loss of the Y chromosome in men and the presence of immune-related tumor suppressor genes in women may also contribute to these sex disparities [31,32]. However, CML-specific data on these mechanisms remains limited and warrants further study.

Furthermore, differences in cancer diagnosis rates may skew sex-related survival statistics. Women more readily and often utilize available health resources compared to men, which may lead to increased diagnoses of asymptomatic cancers [33,34]. This phenomenon may contribute to the observed survival advantages in women, although it does not account for the complex interplay of biological and behavioral factors influencing cancer outcomes.

To address the observed gender disparity in CML mortality, several strategies should be considered. Firstly, targeted health campaigns, especially those aimed at increasing awareness of hematologic malignancies in men, could encourage earlier medical engagement and lead to earlier diagnoses of malignancies. Prior initiatives, such as Movember and Men’s Mental Health Month, have shown promise in promoting men’s health awareness, and similar models could be adapted to increase CML awareness among men. A systematic review analyzed male help-seeking outcomes of mental health media-based campaigns and found that media-based campaigns as a health promotion avenue can positively impact male help-seeking [35]. Secondly, addressing adherence is crucial to improving mortality, as men display lower adherence to long-term therapies, including TKIs. Men may benefit from structured adherence support interventions, such as digital text messaging programs. A randomized trial found that SMS-based adherence interventions improved blood pressure control in patients with hypertension, suggesting a potential model for improving TKI adherence in male CML patients [36]. Lastly, further research is needed to better understand gender-related differences contributing to mortality. The aforementioned differences in biological risk factors, such as immune response and gene expression, warrant further evaluation.

While hormonal, immunologic, and genetic differences may contribute to observed sex-based disparities, these mechanisms remain speculative in the context of CML and are not directly evaluated in this study.

Comparative analysis of CML mortality by demographic factors

When comparing trends in CML mortality across demographic groups from 1999 to 2020, notable differences emerge between race, gender, and age groups. These comparative trends should not be interpreted as evidence of differential biologic disease behavior across demographic groups, but rather as reflections of population-level variation in mortality patterns over time.

Racial disparities in mortality narrowed the most significantly over the period, with mortality rates for both African American and White American populations declining at relatively similar rates, with an AAPC of -4.48% and -3.84%, respectively. Although both groups showed a significant decline, the gap between African American and White American mortality rates remained relatively stable. In contrast, gender disparities also narrowed, although to a lesser extent. While both males and females showed improvement in mortality, the male-female mortality gap decreased by approximately one-third, less than the reduction observed by race.

Age-related disparities, on the other hand, proved to be more persistent. While younger age groups, such as those aged 45-54, saw sharp declines in mortality (AAPC of -6.91%), older populations experienced slower progress. For example, individuals aged 85 and older faced only a slight reduction in mortality over two decades (AAPC of -0.30%). These patterns indicate that while CML mortality has decreased across these three groups, racial disparities improved most markedly, gender disparities narrowed moderately, and age disparities, especially among the elderly, remain largely unchanged.

Limitations

Data in this study were obtained from the CDC WONDER database, a valuable resource for analyzing cancer mortality trends in the United States from 1999 onward. However, this study has many limitations. The database provides aggregated mortality data and cannot provide case-specific information. This limits the ability to analyze individual risk factors such as diet, tobacco use, social isolation, and family history, all of which contribute to the SDOH that affect mortality. Additionally, the dataset does not include information on comorbidities or adherence to therapy, which may contribute to unmeasured confounding and limit the interpretation of observed mortality differences. The database also does not stratify the type of TKI utilized in each case. The database itself lacks many variables suitable for subgroup analysis that contribute to mortality in malignancies, including income status, level of education, and rurality. The data in the CDC WONDER database are subject to the limitations of the reporting systems from which they were drawn. This includes inconsistencies in how different states or regions report mortality data. Regional differences in healthcare access, cancer screening rates, and treatment availability in the data may affect the accuracy of the identified trends.

Some subgroup analyses, particularly among African American populations, included relatively small numbers of events, which may reduce statistical precision and increase variability in subgroup-specific trend estimates. Additionally, the use of CDC WONDER race categories limited to African American and White populations constrains the ability to assess disparities in other racial and ethnic groups.

Although our study focuses on demographic disparities in CML-related mortality, it is important to acknowledge the role of cardiovascular and lifestyle risk factors, including obesity, tobacco use, hypertension, and other comorbid conditions, which directly contribute to survival outcomes. These comorbidities are present in the general population, more commonly in males and older age groups, and may compound the burden of disease in CML patients. The CDC WONDER database does not capture individual-level data, thus limiting our ability to directly assess the contribution of these factors to mortality.

The exclusion of patients younger than 45 years further limits generalizability. However, this restriction was applied because CML is relatively rare in younger populations, and mortality in this age group is uncommon, which may reduce statistical stability and variability in rate estimates. The restriction of the study population to individuals aged 45 years and older reflects a deliberate epidemiologic trade-off to improve rate stability, given the rarity of CML in younger populations, but may limit comparability with other studies that include all adult age groups.

Additionally, because this study relies on ICD-10 coding (C92.1) to identify CML-related deaths, cases in which CML was not accurately coded on death certificates may have been missed, leading to potential under-ascertainment of true CML mortality. Potential misclassification of CML deaths due to reliance on ICD-10 coding (C92.1) may result in under-ascertainment of true cases, which could bias mortality estimates downward.

While confidence intervals are reported for Joinpoint-derived APC and AAPC estimates, the CDC WONDER database does not consistently provide variance estimates for all stratified AAMRs, limiting the ability to generate uniform confidence intervals across all reported AAMR values. While linkage to more granular datasets such as SEER-Medicare or institutional registries would allow for patient-level adjustment and validation of treatment-related effects, such analyses were beyond the scope of this study. Future research integrating multiple data sources may help address these limitations and provide more refined risk estimates.

## Conclusions

The findings of this study highlight persistent disparities in CML mortality across age, gender, and race in the United States. Older adults, men, and African American patients experienced disproportionately higher mortality rates compared to their younger, female, and White counterparts. While the advent of TKI therapy has significantly improved overall survival, these benefits have not been equitably realized. Addressing these disparities requires a multifaceted approach, including improved geriatric assessment and treatment tailoring for older adults, adherence and engagement support for male patients, and expanded access and culturally competent care for African American communities. Further research should focus on understanding the specific social determinants that contribute to these disparities and developing strategies to ensure that all patients have equal access to high-quality cancer care. Recognizing and addressing these disparities may help reduce CML mortality and improve outcomes across all demographic groups.
